# Cells and Materials for Cardiac Repair and Regeneration

**DOI:** 10.3390/jcm12103398

**Published:** 2023-05-10

**Authors:** Reem Saud Alhejailan, Gloria Garoffolo, Vineesh Vimala Raveendran, Maurizio Pesce

**Affiliations:** 1 Cell Biology Department, King’s Faisal Specialist Hospital & Research Center, Riyadh 11564, Saudi Arabia; rhijailan@kfshrc.edu.sa (R.S.A.); vraveendran@kfshrc.edu.sa (V.V.R.); 2 Unità di Ingegneria Tissutale Cardiovascolare, Centro Cardiologico Monzino, IRCCS, 20138 Milan, Italy; gloria.garoffolo@cardiologicomonzino.it

**Keywords:** stem cell therapy, heart, cardiovascular system, scaffold, xenotransplantation, mechano-sensation

## Abstract

After more than 20 years following the introduction of regenerative medicine to address the problem of cardiac diseases, still questions arise as to the best cell types and materials to use to obtain effective clinical translation. Now that it is definitively clear that the heart does not have a consistent reservoir of stem cells that could give rise to new myocytes, and that there are cells that could contribute, at most, with their pro-angiogenic or immunomodulatory potential, there is fierce debate on what will emerge as the winning strategy. In this regard, new developments in somatic cells’ reprogramming, material science and cell biophysics may be of help, not only for protecting the heart from the deleterious consequences of aging, ischemia and metabolic disorders, but also to boost an endogenous regeneration potential that seems to be lost in the adulthood of the human heart.

## 1. Introduction

Cardiovascular diseases (CVD) are the leading cause of hospitalization and death globally, and their incidence is continuously increasing [1,2]. CVD is a broader term which includes disturbances of the heart rhythm, cardiac valve pathologies, genetically driven malformations and, ultimately, peripheral or coronary artery diseases (PAD and CAD), which may culminate, respectively, in critical limb ischemia (CLI) and heart failure (HF).

The use of cells with stem/progenitor characteristics in PAD and CLI has shown a success in clinical translation to a certain extent, given the ability of the chosen cells (e.g., derived from bone marrow, peripheral blood, or cord blood) to promote de novo vasculogenesis by a robust “paracrine effect” [3]. By contrast, the use of a similar setting to regenerate the contractile mass of the heart to compensate the loss of myocytes due to acute/chronic ischemia and/or inflammation has been largely unsuccessful and controversial, due to the absence of resident stem cells that could be activated in situ and/or expanded in vitro prior to being reinjected into the failing hearts [4]. Alternatives to this deficiency have been sought in the use of induced pluripotent stem cells (iPSCs), whose derived cardiomyocytes (CMs) have been employed in preclinical models in small and large animals [5,6], and even in pioneering studies in humans [7]. Although scaled-up systems to produce therapeutic quantities of these cells with enhanced purity have been set, anticipating industrial production [5], several caveats have been expressed due to risks of arrhythmogenicity, incomplete maturation, potential tumor formation, and (at least for allogenic use) immune reactions [8].

Given the lack of endogenous regenerative capacity of the myocardium, the consequence of acute/chronic cardiac ischemia is still considered an irreparable damage leading to progressive replacement of the contractile cells with a stiff, fibrotic scar. Under these conditions, the heart undergoes a series of morphological transformations (e.g., rearrangement of the contractile apparatus and modification of the geometry [9]), changes in mechanical characteristics [10] and reduction of the pumping efficiency [11], representing signs of HF.

With the advent of the tissue engineering [12], the introduction of biological fabrication methods combined with refined systems for cellular genetic manipulation [13] and decryption of mechano-sensitive cues [14] has enabled new strategies to enhance the efficacy of cardiac cell therapy and the elaboration of disease modeling systems using 3D biology approaches [15]. This renews the hope that after the disappointment arising from the failure of the “classical” cell therapy approaches, it will be possible to reach a condition to regenerate the human heart, which still represents the “holy grail” of cardiology.

**Figure 1:**
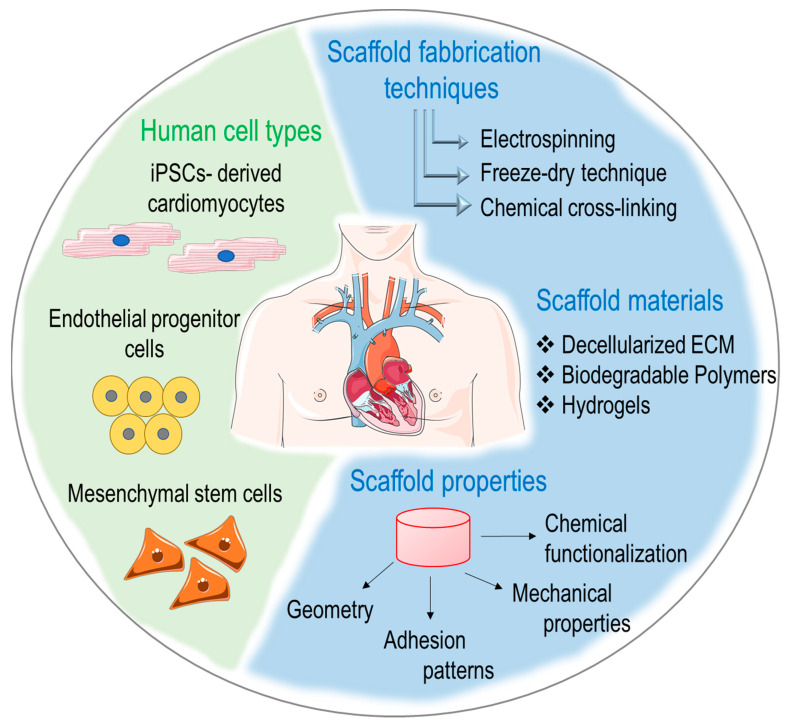
Principal cell types, fabrication techniques and scaffolds employed in current cardiac regenerative medicine and bioengineering.

In an effort to provide a comprehensive view of the actual stage at which studies on regeneration of the human heart are currently, we will illustrate the types of cells that have been historically employed in first-generation cardiac regenerative approaches, the basic principles of cardiac tissue engineering and the new perspectives offered by new materials and biophysical approaches in cardiac repair and regeneration (Figure 1).

## 2. Cell Therapy for Repairing Damaged Myocardium

In first-generation cell therapy, the efficacy testing approach used in animal models and in human translation was quite simple. Based on the paradigm of the bone marrow regeneration, where a single cell can reconstitute the entire set of blood cells into an individual after chemotherapy treatment, it was hypothesized that bulk administration of cells with progenitor characteristics in the infarcted myocardium would lead to the formation of new vessels and/or reconstitution of the contractile mass. Shortly after the beginning of these experiments, it became evident that this approach had low efficiency, and that better methods were necessary to select specific cell populations and to boost the supposed regeneration potential of the cells, if they were to be employed for clinical translation [16].

The modern cellular transcriptomics methods have highlighted an unexpected complexity of myocardial composition with well-established spatial arrangements. For example, Tucker et al., using single cell RNA-seq (scRNAseq) analysis of more than 280,000 single nuclei, identified nine major and over 20 subpopulations of cells within the human heart [17]. Heterogeneity of cells is also increased or significantly affected by myocardial damages. Several published papers have shown, for example, that myocardial ischemia can promote time-dependent expansion of defined populations of cells with antagonistic effects on cardiac inflammation and repair [18,19], as well as variations of physiologic intercellular crosstalk [20], thus hampering myocardial damage. All this diversity in the physiologic cellular composition and the peculiarity of their dynamics in the presence of myocardial damage poses an important question of what cell type could be better employed for myocardial repair, and at what time this ideal cell should be administered to achieve a robust effect. In the following paragraphs we will briefly outline the types and main uses of specific cellular phenotypes that have been employed in first-generation cardiac regenerative medicine approaches (see also Table 1).

### 2.1. Skeletal Myoblasts (SMs)

Satellite cells of the basal lamina in skeletal muscle fibers are the progenitors giving rise to skeletal myoblasts [21,22]. Due to easy accessibility, resistance to ischemic conditions, myogenic capacity and low tumorigenicity, the use of skeletal myoblast as a candidate cell for cardiac regeneration therapy was initiated [23,24,25]. Numerous preclinical studies demonstrated the capability of skeletal myoblasts to differentiate into myotubes, to reduce myocardial fibrosis and ventricular remodeling and to increase the heart pumping efficiency [26,27,28,29]. On the other hand, when these cells were used in clinical trials, despite an improved ejection fraction and enhanced regional wall motion [30,31,32,33,34,35,36], several patients reported ventricular arrhythmias as a side effect [33,34,36,37,38]. Arrhythmia was attributed to the failure to form gap junctions between the engrafted cells and the surrounding myocardium, which resulted in lack of electromechanical coordination [23,39]. The inability of myoblasts to attain a cardiomyocyte-like phenotype has been a significant drawback in their use for cardiac regeneration. The MAGIC trial, which is to date the largest clinical trial using skeletal myoblasts, did not show any beneficial effect on left ventricular function, further highlighting the limitations of this approach [40].

### 2.2. Bone Marrow-Derived Cells

Bone marrow (BM) cells are highly heterogenous, and relatively small populations are represented by cells that can be truly defined as hematopoietic stem cells (HSCs), endothelial progenitor cells (EPCs) and mesenchymal stem cells (MSCs). After the original observations that these cells are mobilized from the bone marrow by ischemia and “home” into hypoxic tissues [41], where they could aid in repairing the damaged vasculature [42], numerous attempts were made in large animals with contrasting results [43,44,45,46,47]. The use of primary BM-derived cells from patients is also controversial due to the potential effects of risk conditions (e.g., diabetes, coronary artery disease) on senescence and epigenetic aging of the cells and, thus, a decrease of their angiogenic potential [48]. As an example, already in 2004, Heeschen et al. noted that bone marrow mononuclear cells (BMMNCs) isolated from patients with ischemic chronic myocardiopathy, irrespective of their similar hematopoietic stem cell content compared to healthy controls, had a significantly reduced neovascularization capacity in a mouse hind limb assay [49]. Furthermore, in a 2006 clinical study in which BMMNCs were injected by an intracoronary route into patients with cardiac ischemia, there was no evidence of improvements in contractility [50].

The purification of selected populations of BM-derived cells, e.g., cells with hematopoietic stem cell (HSCs) characteristics, has been also proposed over time to increase the vascular regeneration potential compared to unselected BMMNCs. One of the first studies demonstrating the effectiveness of BM-derived HSCs was performed in patients with MI, using BM-derived CD133^+^ stem cells injected at the infarct border zone, resulting in improvement in LV function in a three to nine months follow-up period [51]. In patients with ST-elevated myocardial infarction, the mobilization of stem cell populations expressing CD34, CD117, c-met, and/or CXCR4 to peripheral blood was positively correlated with left ventricular ejection fraction and the decrease of NT-proBNP levels and myocardial necrosis markers [52]. More recently, a study showed that autologous injection of CXCR4^+^ HSCs into infarct areas led to the restoration of left ventricle (LV) contractility, as measured by improvement in left ventricle ejection fraction (LVEF; 30 to 50%), stress rate values (from −3/−9% to −18/−22%) and restoration of myocardium vitality as per G-SPECT images [53]. Despite these results, the long-term global improvement in cardiac function using the CD133^+^ or CD34^+^ bone marrow cell fraction remains to be fully demonstrated [54,55].

Another BM-derived population of cells that has been historically employed for vascular regeneration in ischemic tissues is the endothelial progenitor cells (EPCs). These cells play a role in neovascularization by replacing the dysfunctional endothelial cells—the so called “late” EPCs, or by secreting paracrine factors able to stimulate angiogenesis, but not by participating directly to new vessels’ formation—the so-called “early” EPCs [56]. As in the case of HSCs, EPCs can also be mobilized by ischemia, which induces them to proliferate and differentiate under a coordinated action of cytokines, receptors adhesion molecules and paracrine cell signaling mechanisms [56]. The circulating EPCs crosstalk with endothelial cells, cardiomyocytes and cardiac non-myocyte cells also by secreting micro-RNAs (miRNAs) carried in extracellular vesicles (EV) with angiogenic and/or cardioprotective potential [57].

Mesenchymal stem cells (MSCs) are, finally, stem cells of the non-hematopoietic BM stroma, or derivable from adipose tissue, the Wharton Jelly or the umbilical cord, as well as directly from myocardial tissue [58]. MSCs are able to differentiate into various cell types of mesodermal lineage such as osteoblast, adipocytes or fibroblasts. The lack of hematopoietic markers such as, for example, CD45, CD34, CD14/CD11b, CD79a and CD19, makes it distinguishable from hematopoietic stem cells. In addition, since these cells lack HLA-DR, they are immune privileged and therefore suitable for allografting. MSCs can be sourced from BM. In addition to differentiation ability, they secrete many cytokines, growth factors, miRNAs, and extracellular vesicles that make them an ideal choice for treatment of MI [59]. Several preclinical studies have shown promising results with the use of these cells, especially for their paracrine cardioprotective effect [60,61,62,63].

### 2.3. Human Pluripotent Stem Cells

The isolation of embryonic stem cells (ESCs) by Thompson et al. in 1998 from the inner cell mass of human blastocysts [64] added an important tool to the array of potentially employable cells for human regenerative medicine, also in the cardiovascular scenario. Being derived from the embryonic part of the preimplantation blastocyst, hESCs have the ability to differentiate into every cell lineage of the body, thereby offering an inexhaustible source of replacement cells. ESCs have the capability to differentiate into almost any proliferative cell type of three germ layers, including the cardiomyocytes [65]. Further research on activation or inhibition of certain regulatory pathways involved in the fetal heart development such as p38MAPK, Wnt/β-catenin, glycogen synthase kinase 3 (Gsk3), or on the function of prostaglandin I2 (PGI2), helped in setting specialized media to promote the differentiation of ESCs to cardiomyocytes and produce large quantities of differentiated cells for cell therapy purposes [66,67,68]. The possibility of using hESC-derived cardiomyocytes for clinical applications was demonstrated in preclinical studies involving pigs and guinea pigs. Using these models, it was found that the injected cells successfully integrated and functioned as electrophysiologically active cells [69]. The efficacy of ESC-derived cardiomyocytes in repairing myocardium was established in a primate myocardial infarction model [70]. Moreover, ESC-CMs transplanted into the infarcted heart can survive and improve cardiac functions [71]. Despite the early promises, clinical translation of the hESCs is jeopardized by two major shortcomings, the potential formation of teratomas resulting from remaining undifferentiated cells and ethical issues that in many countries prevent the employment of human embryos to derive cells for therapy [8].

The derivation of the so-called induced pluripotent cells (iPSCs) by retroviral transfection of somatic cells using a cocktail of the four transcription factors, Klf4, Sox2, cMyc and Oct3/4 [72], has relieved part of the ethical problems preventing the clinical use of human-derived ES cells, although it has not resolved the problem of the potential tumorigenicity of the differentiated cells derived thereof, as well as of the purity/homogeneity of the derived populations phenotype, and (at least for cardiac therapy) the maturity of the cardiomyocytes [73]. Preclinical studies in large animals have shown an improved contractile function, electrical coupling with host cardiomyocytes in MI hearts at variable times after iPSC-derived cardiomyocytes transplantation [5,74,75]. Despite a relatively positive safety evaluation of the cells when transplanted in patients with severe ischemic heart failure [76], the incompleteness of the differentiation process and the residual possibility of tumor formation still represent a hurdle toward an effective clinical implementation.

**Table 1:** Main cell types employed in cardiac repair.

Cell Type	Advantages	Limitations	Status	Reference
Embryonic Stem Cells	High differentiation potential	Possible tumorigenesis	Preclinical Clinical	[76,77,78]
Induced Pluripotent Stem Cells	Autologous source, high differentiation potential	Risk of tumorigenesis, insufficient differentiation	Preclinical	[74,79]
Skeletal Myoblasts	Contractile properties	Limited engraftment, arrhythmogenic risk	Clinical	[35,36]
Cardiosphere-Derived Cells	Pro-angiogenic and immunomodulatory properties, cardiac-specific	Limited engraftment, inconsistent results	Clinical	[80,81]
Endothelial Progenitor Cells	Pro-angiogenic properties	Limited differentiation potential and engraftment, inconsistent results	Preclinical Clinical	[82,83]
Adipose-Derived Stem Cells	Immunomodulatory and pro-angiogenic properties	Limited differentiation potential and engraftment, inconsistent results	Preclinical Clinical	[84,85]

## 3. Tissue Engineering Strategies to Repair/Regenerate the Failing Heart

In line with the general definition provided in the early 1990s by Langer and Vacanti [12], cardiac tissue engineering can be defined as a fabrication process resulting from combining cardiac-specific or cardiogenic cell types, preferably derived from the patient’s own tissue, with bioactive molecules (e.g., growth factors) and scaffolds manufactured with biocompatible materials [86,87,88]. Particularly important in scaffolds’ design is the three-dimensional arrangement of the basic components (e.g., fibers, polymer blocks) to achieve an adjusted porosity as well as optimized topological and mechanical characteristics. Scaffolds are also functionalized with proteins of the natural extracellular matrix (ECM) to promote physiological adhesion, differentiation, migration and proliferation of the cells of interest [14,89]. For several cardiovascular applications (e.g., vessels or myocardial engineering), scaffolds should be preferentially biodegradable to allow the pre-seeded or the in vivo recruited cells to deposit their own ECM [90,91], although for specific applications such as the engineering of tissues subjected to elevated levels of cyclic strain and compression forces (e.g., the cardiac valves), the use of permanent or semi-permanent scaffolds able to maintain mechanical integrity and resistance may be an advantage [92].

### 3.1. Decellularized ECM for Cardiac Engineering

To date, scaffolds prepared by ECM decellularization demonstrated promising features to mimic cardiac environment [93]. The most striking example of complete heart re-engineering was described in a seminal publication, where Ott and collaborators removed all the cells from rat hearts to reintroduce new cells with cardiac competence restoring, at least in part, cardiac-specific functionalities [94]. Historically, decellularization protocols have been realized using chemical, physical, and/or enzymatic treatments to remove cellular contents from tissues. Chemical methods employ detergents, enzymes, or pH changers to lyse cell membranes [95,96]. Physical methods include freeze–thaw cycles, agitation, mechanical manipulation, or pressure to forcibly remove cells [97]. Enzymatic methods consist, finally, of incubating tissues with proteolytic enzymes such as collagenases and proteases (e.g., trypsin) and/or chelating agents such as ethylenediamine tetra-acetic acid (EDTA) or ethylene glycol tetra-acetic acid (EGTA) [97]. The three methods all have advantages and shortcomings. For example, in the case of osmotic shock by incubation with hypotonic buffers, the subsequent treatment of the tissues with detergents (e.g., SDS) helps to remove lipids and DNA debris, but this could be detrimental for the maintenance of the glycosaminoglycans (GAGs), and even the stability of the collagen [98]. Furthermore, when using proteolytic enzymes, the ability of the cells to repopulate the cardiac cell-free scaffolds could be affected [99].

The efficiency of decellularization protocols is normally evaluated by quantifying the DNA content using spectrophotometric [99] or PCR tests performed on highly repetitive sequences (e.g., microsatellite) [100]. Other controls such as the assessment of GAGs and removal of xenoantigens should be also taken into consideration to monitor the maintenance of an appropriate matrix composition [101], and absence of antigens (i.e., the αGAL) that may cause rejection into the human system [102]. Whenever necessary, mass spectrometry approaches could be eventually used to assess quantitatively the reduction/variation/relative abundance of the main ECM components determined by the decellularization procedure and monitor the reintroduction of cellular proteins by the seeded cells [103,104]. The content in growth factors is a further important feature of scaffolds derived from cardiac tissue to support cell survival and angiogenesis [105]. In case the decellularization procedure reduces their amount, there is still the option to add crucial cytokines (e.g., VEGF, b-FGF, PDGF-BB, IGF-1) to culture media during the recellularization phase to complement their reduced content. A final criterion that should be adopted in validating a decellularization method to generate cardiac tissues is mechanical integrity. To this aim, specific stress/strain curves can be generated using machines for tensile strength determination to measure the mechanical resistance to strain in comparison to the non-decellularized condition. This feature is particularly important, for example, in valve leaflets’ engineering for the necessity to resist up to billion straining cycles [92,104].

### 3.2. ECM-Mimicking and -Derived Materials for Cardiac Engineering

Most of the synthetic materials used in cardiac tissue engineering are biodegradable and have the advantage of being replaced when mature cells produce their ECM. Recently, there has been an emphasis on developing patches for cardiac repair with the use of biodegradable polymers, such as poly (ε-caprolactone) (PCL), poly (glycerol sebacate) (PGS), polyethylene glycol (PEG), poly (l-lactide acid) (PLLA), poly l-lactic-co-ε-caprolactone (PLCL) or poly (lactic-co-glycolic acid) (PLGA) [106]. For instance, to improve spatial cellular orientation, Morgan et al. developed a porous structure using PGS that significantly enhanced cardiac cells’ alignment [107]. In another approach, a tissue-engineered vascular graft made with a mixture of PLLA and PLCL was seeded with the patient’s autologous bone marrow mesenchymal stem cells and used as extracardiac cavo-pulmonary conduit [108]. The graft exhibited a relatively low rate of stenosis (in 28% of patients), with no evidence of aneurysm formation, graft rupture, graft infection, or calcification. In parallel, researchers have also investigated co-polymerization approaches combining artificial and natural polymers for the creation of cardiac constructs with improved compatibility. For example, Feng et al. [109] explored the possibility of creating cardiac patches using a mixture of collagen, chitosan, and various crosslinking methods. After a thorough characterization of the resulting scaffolds, they seeded cardiomyocytes and fibroblasts and concluded that the different mechanical and topological characteristics promoted proliferation and phenotypic maturation of the cells to different extents, demonstrating the finely tuned relationship between spatial arrangement cell mechanics and cellular phenotypic control. Another example of hybrid scaffold manufacturing approaches is the work from Liu and collaborators, in which electrospun scaffolds realized with PCL and natural cardiac-derived proteins (mainly collagen and elastin) mixed in various percentages and seeded with BM-derived HSCs improved cardiac healing after infarction in a dose-dependent manner [110].

Despite the potential advantages, the application of tissue-engineered patches onto the infarcted region to promote cardiac healing could be suboptimal for the risk of incomplete engraftment of cells into the scaffold, thus limiting the therapeutic efficacy. Therefore, with the advancement of hybrid fiber deposition methods, more composite scaffold structures have been designed to include elements that can maintain the mechanical coherence of the patch and, at the same time, exhibit binding sites for the cells. An example is the work of Wee et al., who manufactured a composite scaffold with nanometric/micrometric fibers of collagen and PLGA that had a higher cell retention than conventional scaffolds and performed better in term of cardiac repair after myocardial infarction [111]. Table 2 recapitulates some of the currently employed scaffolds for cardiac tissue engineering.

### 3.3. Combining Cells and Biomaterials for Cardiac Tissue Engineering

The introduction of cells inside decellularized or bioartificial scaffolds can be performed in several ways from a simple manual seeding of the cells to more sophisticated and controllable systems, such as bioreactors, able to favor the mass transport and exchange of nutrients/oxygens and achieve a uniform distribution of the cells inside the constructs [102]. Particularly interesting is the interaction between the seeded cells and the scaffolds that might contribute to evolution of the resulting tissue construct toward a mature condition. In a recellularization study performed using rat-derived cardiac matrix and myocytes derived from human embryonic stem cells, the interaction between the scaffold and the laminin present in the scaffold was recognized to promote maturation of the contractile cells, an enhanced electrical coupling, and an increased sarcomere length, indicative of myocyte functional maturation [117]. In another study, it was found that the ECM obtained from the atrium directed the differentiation of human iPSCs-derived cardiomyocytes toward a preferential atrial phenotype, demonstrating the importance of local matrix factors for the acquisition of mature phenotypes [118]. A combination of cells and decellularized materials offers the advantage of being able to proceed with the personalization of the therapeutic intervention using the patient’s own cells, such as somatic progenitors—e.g., MSCs derived from bone marrow or adipose tissue, or iPSCs—to minimize rejection. This possibility is granted by the removal from the scaffolds of antigens that could induce immune responses against the graft [102], and the full immunological compatibility of the cells [119].

The potential of combining a bioactive extracellular matrix (ECM), whether natural or synthetic, with seeding of cardiomyogenic cells was explored in several studies for myocardial repair in vitro and in vivo. For example, combining a natural ECM hydrogel with brown adipose-derived stem cells (BADSCs) promoted myocardial infarction repair at higher levels compared to cells alone, suggesting a potent cardiomyogenic potential [120]. The combination of macroscopic materials, cells and nanomaterials could also be an advantage for the fine tuning of cellular growth and phenotype. As highlighted in a recent interesting contribution, the inclusion of drug-loaded nanoparticles, carbon nanotubes or of noble metal nanorods could significantly improve the cardiogenic commitment of scaffold-seeded or hydrogel-laden cells [121].

## 4. Xenotransplantation and Cardiac Repair

Despite advances with pharmacotherapies and devices, the only curative option for end-stage heart failure is orthotopic heart transplant (OHT) [122]. However, due to the restricted number of human organs available for transplantation, only few patients can receive cardiac allotransplant each year [123]. The imbalance between the number of hearts necessary for transplantation and the availability of transplantable organs is continuously growing, and this makes the elaboration of alternative solutions extremely urgent. In this respect, xenotransplantation of animal-derived (e.g., pig) hearts may provide an ethical and unlimited resource.

Heart xenotransplantation has been so far addressed in several animal studies, including on non-human primates as the closest species to humans [124], even if recently a gene-edited porcine heart was transplanted in a patient with an end-stage heart failure patient [125,126]. The recipient patient lived for two months receiving high doses of immunosuppressive agents, hence becoming the longest-living human survivor of a cardiac xenotransplant. Importantly, the technical success of this intervention warrants future clinical studies to refine immunosuppression protocols, monitor the possible introduction of infectious pathogens, evaluate the graft function, and assess the physiological performance of the xenotransplant. 

### 4.1. Immunological Challenges

The major challenge with organ xenotransplantation is related to the expression of peculiar tissue antigens that cause acute rejection in humans. One of the most well-known of these antigens is the oligosaccharide galactose-α1,3-galactose (αGal), which is present in all mammals with the exception of humans and non-human primates of the old world [127]. Antibodies recognizing the αGal antigen rapidly activate the complement-mediated immune response, resulting in acute rejection of the transplanted organs due to a tissue degeneration process occurring within minutes to hours by primarily targeting the vascular endothelial cells [128,129]. Graft failure and thrombotic microangiopathy can be also induced in the transplanted organ due to activation of vascular endothelial cells by low levels of anti-αGal antibodies or abnormal coagulation due to incompatibilities in the coagulation/anticoagulation factors [130,131]. Concerning cellular xenotransplantation (e.g., pig-derived cardiac cells in human hearts), although the hyperacute xenograft rejection does not occur, xenogeneic cells can still trigger immune responses, such as the activation of T-cells through direct and indirect pathways. This direct activation is elicited by the binding of T-cell receptors of the recipient to swine leukocyte antigen class I and class II on porcine donor antigen-presenting cells, such as dendritic cells or endothelial cells constitutively expressing CD80/86 [132]. An indirect activation of the immune system is also initiated by the recognition of porcine-specific antigens by major histocompatibility complex (MHC) class II of the recipients. The subsequent T-cell stimulation results in B-cell activation and antibody production, thus mediating humoral xenograft rejection [133]. Several strategies have been elaborated to circumvent the problem of xenoreactive T-cells, such as the repetitive administration of CTLA4Ig (abatacept) and anti-CD40 mAb, as well as anti-CD154 mAb via CD28 [133]. This is, so far, the most successful approach, with positive results for porcine skin [134,135], pancreas [135], and heart transplantation [136].

### 4.2. Genetic Engineering Strategies of Animals and Cells to Ensure Immunological Compatibility

A breakthrough in xenotransplantation has been provided by the generation of α1,3-galactosyltransferase gene-knockout (GTKO) pigs by cloning [137], or nuclear transfer of spontaneously null mutant cells [138], thus opening the way to the transplantation of xenoantigen-free organs. Unfortunately, despite that organs and cells from the GTKO pigs demonstrated prolonged graft survival, xenograft rejection still occurred and was associated with the activation of the innate immune system and coagulation [139]. Therefore, to further improve the outcomes, GTKO pig cells were engineered to express human complement-regulatory proteins, as investigated in vitro [140] and in vivo [141]. In the meantime, other porcine antigens, e.g., the N-glycolylneuraminic acid (NeuGc), were found to induce natural antibodies in humans, thereby explaining the suboptimal results obtained with the heterotropic transplantation of GTKO pig tissues [142]. The disappointing results of single-knockout animals were at least in part corrected by the generation of pigs with multiple knockouts. For example, in 2015 double-knockout pigs lacking the genes for the N-glycolylneuraminic acid and galactose α-1,3-galactose to prevent the adverse effects of the antibody–antigen interactions were generated [143]. The addition of a further mutation in the β1,4 N-acetylgalactosaminyltransferase (Sda) (β4GalNT2) gene to the previous double-knockout background further increased compatibility [144]. In another study, pigs overexpressing pCTLA4-Ig were produced both from a wild-type and GTKO background to address T cell-mediated immune response. However, these pigs exhibited reduced humoral immunity, which made necessary the use of antibiotics to maintain their health [145]. An alternative way to that of generating mutant animals for combinations of xenoantigens involves, finally, the overexpression of immune regulatory ligands. For example, by overexpressing the human programmed cell death ligand 1 (PD-L1), known to suppress the proliferation of human CD4 T-cells and enhance regulatory T-cells coupled expansion with increased interleukin-10 production, a focus was made on the potential of using human transgenes to foster tolerance of other cell and tissue xenotransplants [146].

Taken together, these results suggest that an ideal candidate as a xenogenic donor of hearts for human transplantation might be a pig carrying multiple xenoantigen knockout mutations, and engineered to expresses low levels of human complement regulatory proteins and human coagulation regulatory proteins. In this respect, the expression of human transgenes, such as heme oxygenase 1 and CD47, might be valuable for improving graft survival due to general anti-inflammatory effects and the suppressive effect on monocyte and macrophage function [147].

## 5. Harnessing Cell Mechano-Sensation to Repair/Regenerate the Heart

Cells are continuously exposed to mechanical stimuli deriving from the surrounding ECM or from neighboring cells. The term “mechano-transduction” indicates the ability of cells to convert these physical signals into intracellular signaling and biological responses affecting cell phenotype and functions. This mechanism requires the involvement of specific molecules expressed at the cell membrane that act as mechano-sensors, such as integrins, stretch-activated ion channels or G protein-coupled receptors [148]. Due to their phenotypic control in differentiated cells and cells with progenitor characteristics [14], the integration of mechanical cues in the current design of advanced cardiac engineering is becoming a crucial component.

### 5.1. From Force Decryption to Intracellular Signaling

The force sensing starts from the focal adhesion, a large complex which contains several specialized cytoplasmic proteins that communicate directly with the cytoskeleton. In particular, in mature focal adhesions, the transmembrane proteins integrins are connected with ECM proteins through the extracellular head domains and with actin cytoskeleton through the activation of talin and vinculin [149]. Additionally, several actin-regulating proteins are involved in integrin-dependent force transmission [150]. During the initial adhesion formation, integrins are linked to the cytoskeleton by talin, then α-actinin competes with talin for the binding to integrin tails. At this point, α-actinin links actin to integrins and transmits forces to the ECM to complete adhesion maturation. The transduction of the signal subsequently occurs through contraction/alteration of the cytoskeleton that activates downstream signaling. In particular, it has been demonstrated that the integrin-focal adhesions complex modulates the Hippo pathway and its nuclear transducers, YAP (Yes-associated protein) and TAZ (transcriptional coactivator with PDZ-binding motif, also known as WWTR1) [150]. The activity of the YAP/TAZ complex is related to the mechanical status of the cells and it is controlled by the geometric constraints of the cells, or stiffness of the adhesion substrate. In particular, the YAP/TAZ duo is translocated from the cytoplasm (where it is transcriptionally inactive) to the nucleus (where it is active) depending on mechanically activated phosphorylation status controlled by the Hippo kinase pathway [151]. Increased substrate stiffness or cell spreading shift the equilibrium toward a nuclear localization of the complex, which is then free to interact with TEA domain (TEAD) DNA binding proteins to regulate gene transcription [152]. By contrast, in cells subjected to low forces such as by adhering onto substrates with low stiffness, or in the occurrence of less spread shapes, YAP/TAZ are phosphorylated by Hippo kinases and this causes an increase in proteasomal degradation and consequent reduction of the transcription factor in the nucleus [152]. In addition to the reversible control of the degradation by the kinase pathway, the YAP/TAZ duo can be forced to enter the nucleus by the stress-fibers-dependent physical deformation of the nuclear lamina and the consequent opening of the nuclear pores [152]. Transmission of forces from cytoskeleton to the nucleus is particularly relevant, not only to allow the trafficking in and out of transcription factors, but also to alter the topology of the nucleus by affecting the activation status of the chromatin and promoting gene transcription [153]. Indeed, interfering with actin cytoskeleton polymerization using pharmacological inhibitors not only affects YAP nuclear localization, but also determines a relaxation of the chromatin resulting in a decrease nuclear stiffness and a reduction of canonical YAP targets transcription [154].

The case of the YAP/TAZ complex is not unique; in fact, other transcription factors involved in cardiac biology are also mechanically regulated (Table 3). One example is the pathway controlled by the serum response factor (SRF) transcriptional activator and the co-activator myocardin-related transcription factor (MRTF), which requires Rho-dependent actin polymerization to translocate into the nucleus. MRTF interact with SRF controlling the transcription of genes related to ECM production and smooth muscle differentiation [155]. Opposite to the YAP/TAZ-dependent pathway, interfering with MRTF nuclear localization using actin cytoskeleton inhibitors prevents cell differentiation and the onset of pro-pathological conditions [156]. Mechanical stimulation controls, finally, the transcriptional activity of β-catenin by promoting the phosphorylation of the protein at a specific tyrosine residue implicated in the interaction with E-cadherin. The consequent reduction in the interaction with the adhesion molecule favors the phosphorylated β-catenin nucleus translocation where, in turn, it activates the transcription of WNT-responsive genes [156].

### 5.2. Mechanotransduction in Heart Physiology and Pathology

Mechanotransduction plays an important role in driving cardiac morphogenesis. Changes in ECM stiffness in the heart primordium promote the initial beating of the embryonic myocytes by opening the mechanosensitive Ca^2+^ channels, before the onset of electromechanical coupling [158]. The stiffness of the surrounding ECM also plays a role in the switch between fetal proliferative and early postnatal hypertrophic growth. In fact, the stiffening of the ECM surrounding the proliferating myocytes promotes sarcomere organization, allowing cardiomyocytes to contract with a higher force [159]. By contrast, the softening of the cardiac matrix by treating with collagenases suppresses beating by altering cytoskeletal conformation, preventing sarcomere assembly into cardiomyocytes [160], and extending the cardiomyocyte proliferation phase in vivo [161].

At early postnatal and adult stages, cardiomyocytes do not proliferate and cardiac growth is supported by the expansion of cellular size (hypertrophy). This event is characterized by an apparently irreversible cardiomyocyte differentiation due to the inability to complete cytokinesis. While the inhibition of cell division is essential to prevent uncontrolled growth of the myocardium, it also limits the ability of the cardiomyocytes to re-enter the cell cycle and regenerate the injured heart. The fine modulation of ECM rigidity could play a major role in this. This evidence is supported by experiments in zebrafish, in which a transient softening of the ECM allows the dedifferentiation of cardiomyocytes sarcomere structure, and thus the regeneration of cardiac tissue by reactivation of myocytes growth [162]. The relationship between matrix stiffness and the reversible differentiation of cardiomyocytes has been further demonstrated by experiments performed on low-stiffness gels where a higher proliferation was maintained [163]. This finding has an interesting readout in patients implanted with left ventricle assist devices, where the mechanical unloading of the ventricular tissue seems to be accompanied by the restoration of myocytes’ proliferation [164]. Altogether, these demonstrations suggest that a fine tuning of mechanical and viscoelastic characteristics of the extracellular environment could be crucial to promote re-entry of the myocytes into the cell cycle, thus giving rise to an authentic cardiac regeneration program [164].

### 5.3. Mechanical-Dependent Pathologic Signaling

Besides instructing the correct differentiation of stem and progenitor cells for engineering cardiac tissues, mechanical proprieties of the matrix have also been shown to drive the differentiation of cardiac-resident cells towards pro-pathological cell fates [165]. This is relevant for progression of the maladaptive myocardial remodeling (one of the processes predisposing to HF) due to inflammation and an excessive production of ECM proteins by the so-called “myofibroblasts” [166]. Mechanical cues are emerging as a major driver of cardiac myofibroblast activation. In a recent work [166], for example, we have shown that after myocardial infarction, cardiac stromal cells are directly exposed to incremental strain/compression forces that induce the activation of the YAP-dependent transcriptional pathway, leading to myofibroblast activation and abundant collagen deposition. Blockading of the YAP/TAZ complex using verteporfin, a drug that interferes with the binding of YAP to TEADs, attenuates cardiac fibrosis and remodeling. It was interesting to note that inhibition of the mechanical pathway by treating cells with the drug overrode the TGFβ-dependent myofibroblasts’ activation [167]. While this revealed a crosstalk between mechanical cues and the humoral control of fibrosis, it also showed that mechanical cues are prevalent in the pathological vs. physiological differentiation of cardiac fibroblasts. The cooperation between the Hippo/YAP pathway and TGFβ signaling was also revealed by another study published by us. In this study, we showed that interfering with the YAP/TAZ transcriptional function was sufficient to revert the matrix compaction ability of human cardiac fibroblasts primed with TGFβ [168], again confirming the relevance of mechanical cues for pathology progression.

## 6. Conclusions

Since the first mention of the tissue engineering concept by Langer and Vacanti in 1993 [12], significant efforts have been made to provide innovative solutions for highly impacting pathologies of the cardiovascular system, such as heart failure, valve calcification and coronary artery disease. Compared to the originally proposed concept, the implementation of stem/progenitor cells in the engineered tissues design must be adapted to include crucially emerging additional components such as geometry, adhesion patterns, mechanical properties and chemical functionalization. These crucial elements are, in fact, necessary to achieve the local instruction and fine tuning of cellular phenotype to increase reparative potential and, potentially, therapeutic efficiency. While the increase in the complexity of the replacement tissues design parallels the evolution of scaffolds fabrication methods, the knowledge on molecular specification of the cells in complex tissues (e.g., by single cell/nuclei gene expression analysis) will help to assess the most suitable cell types to enhance the efficiency of the repair/regeneration processes. Only through this advancement will it be possible to acquire a real translational potential to improve the constantly increasing challenges represented by pathologies of the heart.

## Figures and Tables

**Table 2:** Scaffolds employed in cardiac tissue engineering.

Scaffold	Technique	Results	Year	Reference
Conductive nanofiber scaffold (polypyrrole hydrogel/chitosan/polyethylene oxide)	Electrospinning	Cell adhesion, growth and proliferation, conductive nanofiber scaffolds appropriate to use in cardiac tissue engineering.	2021	[112]
Polypyrrole scaffold coated with silk fibroin	Electrospinning	Mimic of myocardium fibrils, resemble mechanical properties to the native myocardium, good electrical conductivity for cardiomyocytes, and support CM contraction.	2021	[106]
Composite of cardiac ECM with alginate and chitosan	Freeze-dry technique	Very high swelling rate and porosity, stability in PBS solution, improving of the tensile strength, proliferation of human MSC inside the pores, high marker cTnT expression	2020	[113]
(Collagen/carbon nano tubes/chitosan/gold nanoparticles) Injectable hydrogel	Chemically cross-linking	Non-toxic, optimum potential as a new biomaterial for cardiac tissue engineering applications.	2020	[114]
Alginate scaffolds functionalized with magnetite nanoparticles	Freeze-dry technique	Magnetic alginate scaffolds exposed to an alternating magnetic field create stimulating microenvironments for functional tissue engineering	2021	[115]
Cardiac ECM-chitosan-gelatin composite	Freezing and lyophilization	High pore size, biodegradable and biocompatible, high cell survival and proliferation.	2019	[116]

**Table 3:** Mechanosensitive pathways of cardiac relevance.

Pathway	Effectors	Functions	References
Hippo	YAP/TAZ	Drives myofibroblast activation and promotes collagen deposition	[154]
Rho-A	MRTF-A	Interacts with SRF to regulate the transcription of genes involved in ECM production	[155]
Wnt	β-Catenin	Translocates into the nucleus to initiate the transcription of cardiac-related genes	[157]

## Data Availability

Not applicable.
